# Low rates of prescribing alcohol relapse prevention medicines in Australian Aboriginal Community Controlled Health Services

**DOI:** 10.1111/dar.13708

**Published:** 2023-07-09

**Authors:** Gemma Purcell‐Khodr, James H. Conigrave, K. S. Kylie Lee, Julia Vnuk, Katherine M. Conigrave

**Affiliations:** ^1^ NHMRC Centre of Research Excellence in Indigenous Health and Alcohol, Central Clinical School, Faculty of Medicine and Health The University of Sydney Sydney Australia; ^2^ School of Rural Health, Faculty of Medicine and Health The University of Sydney Dubbo Australia; ^3^ Institute for Positive Psychology and Education Australian Catholic University Sydney Australia; ^4^ Edith Collins Centre for Translational Research in Alcohol Drugs and Toxicology, Drug Health Services Sydney Local Health District Sydney Australia; ^5^ National Drug Research Institute Curtin University Perth Australia; ^6^ Burnet Institute Melbourne Australia; ^7^ Centre for Alcohol Policy Research La Trobe University Melbourne Australia; ^8^ Aboriginal Health Council of South Australia Adelaide Australia; ^9^ Adelaide Rural Clinical School, Faculty of Health and Medical Sciences The University of Adelaide Adelaide Australia; ^10^ Drug Health Services Royal Prince Alfred Hospital Sydney New South Wales Australia

**Keywords:** Aboriginal Community Controlled Health Services, alcohol dependence, alcohol use disorder, primary care, relapse prevention medicine

## Abstract

**Introduction:**

Alcohol dependence is a chronic condition impacting millions of individuals worldwide. Safe and effective medicines to reduce relapse can be prescribed by general practitioners but are underutilised in the general Australian population. Prescription rates of these medicines to Aboriginal and Torres Strait Islander (First Nations) Australians in primary care are unknown. We assess these medicines in Aboriginal Community Controlled Health Services and identify factors associated with prescription.

**Methods:**

Baseline data (spanning 12 months) were used from a cluster randomised trial involving 22 Aboriginal Community Controlled Health Services. We describe the proportion of First Nations patients aged 15+ who were prescribed a relapse prevention medicine: naltrexone, acamprosate or disulfiram. We explore associations between receiving a prescription, a patient AUDIT‐C score and demographics (gender, age, service remoteness) using logistic regression.

**Results:**

During the 12‐month period, 52,678 patients attended the 22 services. Prescriptions were issued for 118 (0.2%) patients (acamprosate *n* = 62; naltrexone *n* = 58; disulfiram *n* = 2; combinations *n* = 4). Of the total patients, 1.6% were ‘likely dependent’ (AUDIT‐C ≥ 9), of whom only 3.4% received prescriptions for these medicines. In contrast, 60.2% of those who received a prescription had no AUDIT‐C score. In multivariate analysis, receiving a script (OR = 3.29, 95% CI 2.25–4.77) was predicted by AUDIT‐C screening, male gender (OR = 2.24, 95% CI 1.55–3.29), middle age (35–54 years; OR = 14.41, 95% CI 5.99–47.31) and urban service (OR = 2.87, 95% CI 1.61–5.60).

**Discussion and Conclusions:**

Work is needed to increase the prescription of relapse prevention medicines when dependence is detected. Potential barriers to prescription and appropriate ways to overcome these need to be identified.

## 1 INTRODUCTION

Unhealthy alcohol use [1] is a major cause of preventable disease, early mortality [2] and a causal factor in family and community violence [3]. Unhealthy use includes hazardous, harmful and dependent alcohol use [4, 5]. For Aboriginal and Torres Strait Islander (First Nations) Australians, alcohol‐related harms and hospitalisations are two to three times greater than in the general Australian population [6, 7]. This is in part due to the prevalence and context of episodic heavy drinking [8, 9, 10]. That drinking occurs on the background of grief, loss, intergenerational trauma and socioeconomic marginalisation as a result of colonisation [11, 12]. Alcohol dependence is at the severe end of the drinking spectrum (comparable to moderate to severe alcohol use disorder in the Diagnostic and Statistical Manual of Mental Disorders [13]). It is characterised by a strong internal compulsion to consume alcohol with impaired ability to control one's use. Among First Nations Australians, the prevalence of alcohol dependence is similar to that of the general Australian population (2.2% vs. 1.4%, aged 16+) [14, 15]. Harms to individuals, their families and communities can be greater in dependence [9]. To help address alcohol dependence among First Nations Australians, accessible and effective treatments are needed.

Primary care services are the first point of contact with the health‐care system for most patients and provide care across the lifespan. Patients generally have long‐standing relationships with primary care doctors, and these relationships can also include their families and connections to the wider community [16]. As such, primary care doctors can develop strong rapport with patients and play a key role in screening for unhealthy alcohol use, including detecting dependence and utilising existing treatments [17].

For patients who meet the criteria for alcohol dependence, there are three medicines approved for use in Australia to help prevent relapse after stopping drinking—naltrexone, acamprosate and disulfiram [18]. These medicines can be prescribed by general practitioners (GP). The government subsidises the cost of naltrexone and acamprosate via the Pharmaceutical Benefits Scheme [19]. Disulfiram is not subsided and costs approximately $80–90AUD per month [20]. Despite the effectiveness (and cost‐effectiveness) of relapse prevention medicines [21], they remain under‐prescribed compared with the level of need. Between 2009 and 2013, it was estimated that only 3% of Australians with alcohol dependence were prescribed naltrexone or acamprosate [19, 22]. No data are available on prescriptions for disulfiram. Little is known about Australian First Nations peoples' access to relapse prevention medicines. However, this population tends to have lower access to prescription medicines on average compared to their non‐Indigenous counterparts. Between 2016 and 2017, the average Pharmaceutical Benefits Scheme expenditure per First Nations Australian was 29% of the amount spent for each non‐Indigenous Australian [23].

There are promising results from trials of naltrexone and disulfiram among other First Nations peoples, although the study designs limit the strength of their findings. Disulfiram was trialled in two separate (uncontrolled) studies in Navaho communities. One study reported decreased binge drinking and increased sober periods at 18 months [24]. The second study reported 78% reduction of alcohol‐related incarcerations and just under half the participants (*n* = 50/115) were abstinent at 12–24 months [25]. These reports were published more than 50 years ago, and the detail provided does not allow conclusions on the acceptability of this medication to these First Nations peoples. A recent randomised controlled trial in Alaska reported that naltrexone was associated with a significant reduction in alcohol‐related adverse consequences when compared with placebo. On the other hand, in this 3‐arm trial, naltrexone combined with sertraline was not significantly better than placebo, though sample size (*n* = 68) and hence, power, was limited [26].

Aboriginal and Torres Strait Islander Community Controlled Health Services (ACCHS) are primary care services managed and delivered by First Nations communities across Australia [27]. ACCHSs deliver holistic, culturally informed care in over 300 clinics across urban, regional and remote settings. Understanding prescribing rates for alcohol relapse prevention medicines and potential patient and service factors associated with their prescription, could help to optimise uptake and inform use of the medicines. In the current study, we aimed to: (i) determine the extent of prescribing of alcohol relapse prevention medicines in 22 ACCHSs over a 12‐month period; and (ii) investigate whether demographic factors or AUDIT‐C screening were associated with prescription of these medicines.

## 2 METHODS

### 2.1 Overview

This is a secondary analysis of routinely collected data, provided by 22 ACCHSs as part of the baseline for a cluster‐randomised trial [28]. That study examined the effectiveness of a model of multi‐component, service‐wide and collaborative support designed to increase screening and treatment for unhealthy alcohol use (trial registration number: ACTRN12618001892202) [29]. Here, we explore the rates and correlates of prescribing alcohol relapse prevention medicines among First Nations Australians using logistic regression.

### 2.2 Study design

This study presents a cross‐sectional analysis of prescription of alcohol relapse prevention medicines (naltrexone, acamprosate or disulfiram) at baseline across the entire sample (intervention and wait‐list control services).

### 2.3 Setting

Twenty‐two ACCHSs across six states and territories of Australia (New South Wales, Queensland, South Australia, Victoria, Western Australia and Northern Territory). The service sites include urban (*n* = 10), regional (*n* = 5) and remote (*n* = 7) areas.

### 2.4 Data extraction

All services provided routinely collected data for a 1‐year period prior to the intervention start date, that is from 29 August 2016 to 28 August 2017. Deidentified data were provided on patients aged 15 years and over, extracted from the ‘Communicare’ practice software system [30]. Individual patient records were linked with an identification (ID) variable.

### 2.5 Measurements

#### 2.5.1 Demographics

Patient age and gender were recorded by services. Remoteness (urban, regional or remote) was determined based on service location using the Australian Statistical Geography Standard Remoteness Structure [31].

#### 2.5.2 Alcohol Use Disorders Identification Test‐ Consumption questions

The Alcohol Use Disorders Identification Test (AUDIT) is a commonly used alcohol screening tool that can be used to identify hazardous alcohol use and alcohol use disorders (including dependence). The shortened 3‐item tool (AUDIT‐C, i.e. AUDIT ‐ Consumption) has been validated for use with First Nations Australians in comparison with the full AUDIT screen (10‐item) [32]. The full AUDIT has been found to correlate well with another measure of alcohol consumption in one remote Australian Aboriginal community [33, 34]. For this study, AUDIT‐C cut‐off scores were selected to maximise sensitivity and specificity based on the literature. AUDIT‐C scores were classified as ‘risky’ at ≥3 for females and ≥4 for males (sensitivity 0.73, specificity 0.91; sensitivity 0.86, specificity 0.89; respectively) [35]. AUDIT‐C scores of 9 or above were considered to indicate likely dependence (sensitivity 0.87, specificity 0.94) [32]. Patients with no AUDIT‐C recorded in their patient files were excluded from analyses relating to the score.

#### 2.5.3 Relapse prevention medicine prescription

Communicare patient software automatically records prescription of naltrexone, disulfiram or acamprosate. For each relapse prevention medicine, we used binary variables to describe whether patients were prescribed it at least once (1) or not at all (0) during the reference period.

### 2.6 Analysis

We merged data into a single table using the statistical software ‘R' [36] and the data.table library. Data were grouped so that there was a single observation for each patient over the 12‐month reference period. We coded age into four groups (15–24, 25–34, 35–54 and 55+ years) so that nonlinear relationships between age and outcome variables could be observed. For patients with multiple AUDIT‐C scores, the median was used in analyses.

We calculated the number of individuals who were prescribed any one of the three alcohol relapse prevention medicines as a percentage of the total patient population, and compared this across the 22 services for the 12‐month baseline period.

Bivariate logistic regressions were used to investigate the association between potential predictor variables and prescription of any alcohol relapse prevention medicine (dependent variable). Predictor variables included gender, age, service remoteness and AUDIT‐C variables (binary ‘screened’ vs. ‘not screened’; continuous AUDIT‐C scores; and binary ‘likely dependent’ vs. ‘not likely dependent’). Then, three multivariate logistic regressions were used to study the effects of AUDIT‐C screening on prescribing. Pilot analyses demonstrated that findings were not clustered by service (intraclass correlation coefficient 0). Accordingly, we did not include random effects in our logistic regression models. We fit these models using the ‘glm’ function from the stats library in R [36].

### 2.7 Ethics

Ethical approval was obtained from eight ethics committees across Australia: Aboriginal Health and Medical Research Council of NSW Ethics Committee (NSW; project 1217/16), Central Australian Human Research Ethics Committee (project CA‐17‐2842), Human Research Ethics Committee of Northern Territory Department of Health and Menzies School of Health Research (project 2017–2737), Central Queensland Hospital and Health Service Human Research Ethics Committee (project 17/QCQ/9), Far North Queensland Human Research Ethics Committee (project 17/QCH/45‐1143), The Aboriginal Health Research Ethics Committee, South Australia (SA; project 04‐16‐694), St Vincent's Hospital Melbourne Human Research Ethics Committee (project LRR 036/17) and Western Australian Aboriginal Health Ethics Committee (WA; project 779).

### 2.8 Australian First Nations contributions and community participation

The overarching study has significant contributions from Australian First Nations researchers, services and communities [29]. First Nations staff of two state‐wide umbrella agencies for ACCHSs (in South Australia and New South Wales) helped formulate the research question and study design of the overarching trial. Kristie Harrison, a Wiradjuri woman, played a key role in recruiting services, and in other aspects of the overall study. The present sub‐study is led by an Australian First Nations (Gundungurra) author (GK).

## 3 RESULTS

### 3.1 Sample characteristics

Across the 22 ACCHSs, there were 52,678 unique patient observations in the 12‐month reference period. The sample size equates to 6.6% of the First Nations Australian population [10]. Patient demographics, AUDIT‐C screening results and prescriptions by service remoteness are reported in Table 1. Females represented just over half (56.1%) of all patients. The mean age and proportion of female patients were similar across services in urban, regional and remote areas.

**TABLE 1:** Patient demographics, AUDIT‐C scores and alcohol relapse prevention medicine prescription by ACCHS remoteness in the 1‐year baseline period.

Characteristic	Urban	Regional	Remote	Total
Female patients	56.64	55.66	55.95	56.14
Mean age (SD)	36.96 (16.14)	38.84 (16.82)	37.45 (15.9)	37.54
Total number of patients attending services	18,720	10,326	23,632	52,678
Number of patients with:				
A screen with AUDIT‐C (%)^a^	2800 (14.9)	947 (9.17)	5314 (22.4)	9061 (17.2)^d^
Risky AUDIT‐C (%)^b^	1084 (38.7)	566 (59.8)	2197 (41.3)	3847 (7.30)^d^
Likely dependent AUDIT‐C (%)^c^	228 (8.14)	143 (15.1)	483 (9.08)	854 (1.62)^d^
Prescriptions issued *n* (% total)^e^	70 (57.3)	13 (10.6)	39 (31.9)	122^f^
Naltrexone (*n*)	39	8	11	58
Acamprosate (*n*)	30	4	28	62
Disulfiram (*n*)	1	1	0	2

Abbreviations: ACCHS, Aboriginal and Torres Strait Islander Community Controlled Health Services; AUDIT‐C, Alcohol Use Disorders Identification Test‐Consumption.

^a^ Percentage of patients screened with AUDIT‐C from total number of patients attending services.

^b^ Percentage of screened patients returning a ‘risky’ score. AUDIT‐C cut off of ≥3 for females and ≥4 for males was used.

^c^ Percentage of screened patients returning a ‘likely dependent’ score (a subset of risky drinkers). AUDIT‐C cut off ≥9 for females and males was used.

^d^ Percentage of the total patient population.

^e^ Percentage of total number of prescriptions issued.

^f^ Some patients received a prescription for more than one type of medicine (*n* = 4).

### 3.2 AUDIT‐C screening

Under a fifth of patients (17.2%) were screened with AUDIT‐C during the 12‐month reference period (Table 1). Of the patients with an AUDIT‐C score, 42% were classified as ‘risky drinkers’ and 9.4% as ‘likely dependent’ (9.4% is a subset of the risky drinkers). Of the total patient sample, this equated to 7.3% classified as ‘risky drinkers’ and 1.6% as ‘likely dependent’. Males tended to have higher AUDIT‐C scores than women (mean score of 4.02 vs. 2.4; *p* = 0.001). Patients from regional services were more likely to have AUDIT‐C scores indicating likely dependence than patients from other areas (odds ratio [OR] 1.49 [95% confidence interval [CI] 1.32, 1.69]).

### 3.3 Prescription rates

**FIGURE 1:**
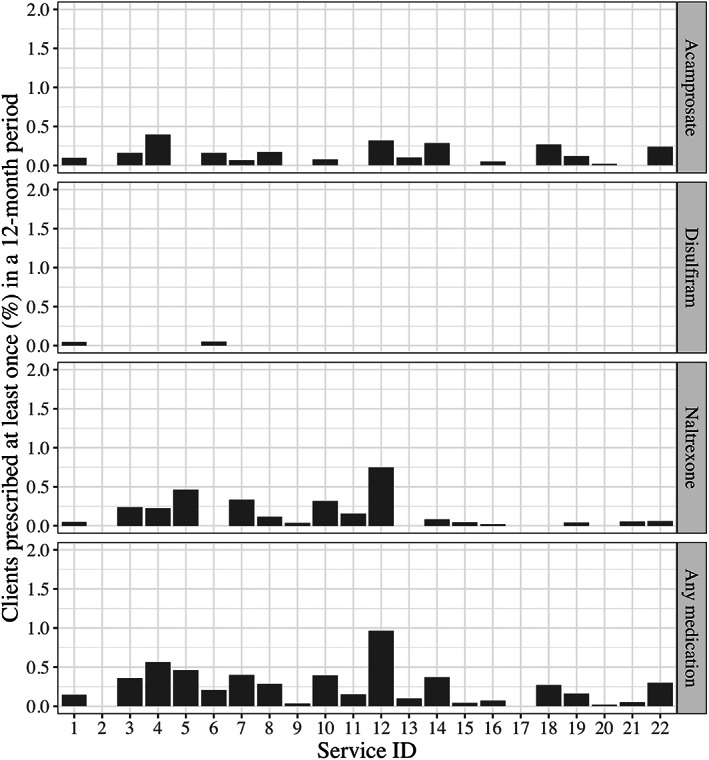
Percentage of patients who received at least one alcohol relapse prevention medicine prescription at 22 Aboriginal and Torres Strait Islander Community Controlled Health Services over the 12‐month baseline period. Calculated as a percentage of the total number of patients who accessed each service during the 12‐month baseline period. In total 118 patients received at least one prescription of any of these medicines.

In total, 118 patients (0.2%) received at least one prescription for a relapse prevention medicine. Of the three medicines, acamprosate was prescribed most (*n* = 62) followed by naltrexone (*n* = 58) and then disulfiram (*n* = 2). Four patients received prescriptions for more than one kind of relapse prevention medicine (Table 1). Overall, rates of prescription were consistently low across all services (Figure 1) with the highest rate in a service being less than one in 100 patients (0.97%). Two services did not record any prescriptions (Figure 1). Patients attending urban services were almost three times more likely to be prescribed a medicine compared to those attending regional services (OR 2.87 [1.61, 5.60]). Patients at remote services were not significantly more or less likely to be prescribed relapse prevention medicines than patients at regional services (OR 1.42 [95% CI 0.77, 2.94]).

The majority (*n* = 71, 60.2%) of patients who received a prescription had no recorded AUDIT‐C score and 18 patients (15.3%) received a prescription while their AUDIT‐C score was not suggestive of dependence (i.e., <9). On the other hand, of the patients with a likely dependent AUDIT‐C score, only 3.4% (*n* = 29) received a prescription.

### 3.4 Predictors of relapse prevention medicine prescription

Males were more than twice (OR 2.2 [95% CI 1.54, 3.28]) as likely to be prescribed any of the medicines compared to females (Table 2). In the multivariate regression which included AUDIT‐C screening status (screened, not screened) as a predictor, male gender remained an independent predictor of prescription (OR 2.24 [1.55, 3.29]). However, after accounting for AUDIT‐C scores (continuous), age and remoteness in a multivariate regression, being male was no longer a significant predictor of receiving a prescription (OR 1.1 [95% CI 0.61, 2.14]).

**TABLE 2:** Logistic regression analyses of AUDIT‐C screening data as a predictor of being prescribed an alcohol relapse prevention medicine when accounting for demographics.

Variables	Unadjusted OR^a^ [95% CI]^b^	Adjusted OR [95% CI] multivariate regressions^c^		
		Screened with AUDIT‐C (yes/no)	AUDIT‐C score (continuous)	Screened likely dependent (yes/no)
Screened with AUDIT‐C (yes/no)	3.20 [2.20, 4.61]***	3.29 [2.25, 4.77]***	–	–
AUDIT‐C score (continuous)	1.54 [1.39, 1.71]***	–	1.48 [1.35, 1.65]***	–
Likely dependent (yes/no)	15.99 [8.92, 29.44]***	–	–	12.78 [6.93, 24.21]***
Male gender^d^	2.24 [1.54, 3.28]***	2.24 [1.55, 3.29]***	1.12 [0.61, 2.14]	1.42 [0.77, 2.71]
Age^e^, years				
25–34	7.13 [2.75, 24.26]***	7.46 [2.88, 25.41]***	2.33 [0.53, 16.02]	2.71 [0.62, 18.59]
35–54	14.53 [6.04, 47.66]***	14.41 [5.99, 47.31]***	6.44 [1.92, 40.03]*	7.74 [2.32, 48.00]**
55+	5.88 [2.13, 20.62]**	5.96 [2.16, 20.91]**	4.34 [0.99, 29.90]	4.19 [0.96, 28.73]
Service remoteness^f^				
Remote	1.42 [0.77, 2.84]	1.12 [0.60, 2.26]	1.24 [0.50, 3.77]	1.04 [0.42, 3.16]
Urban	3.09 [1.73, 6.00]***	2.87 [1.61, 5.60]***	2.36 [0.94, 7.18]	2.05 [0.82, 6.26]

Abbreviations: AUDIT‐C, Alcohol Use Disorders Identification Test‐Consumption; CI, confidence interval; OR, odds ratio.

^a^ The unadjusted odds ratios were derived from the bivariate regressions.

^b^ *p < 0.05, **p < 0.01, ***p < 0.001.

^c^ The adjusted odds‐ratios were derived from multivariate logistic regressions which accounted for demographic variables and the AUDIT‐C variable listed in that column.

^d^ The reference group was female patients who had a log‐odds of being prescribed of −6.53.

^e^ The reference group was those aged 15–24 years who had a log‐odds of being prescribed of −8.16.

^f^ The reference group were regional services whose patients had a log‐odds of being prescribed of −6.76.

In both the unadjusted and adjusted models (Table 2), young people aged 15–24 years were less likely to be prescribed than other age groups. When accounting for AUDIT‐C score, only 35–54 year olds had higher odds of being prescribed relapse prevention medicines than young people (15–24 year olds).

Being screened with AUDIT‐C was consistently a significant predictor of prescription. Accounting for demographics, patients who were screened with AUDIT‐C (regardless of their score), were over three times more likely to be prescribed a medicine than those who were not screened (OR 3.29, [2.25, 4.77]) (Table 2). Patients with a likely dependent AUDIT‐C score were almost 13 times more likely to be prescribed a medicine than those with a non‐dependent score (OR 12.78, [6.93, 24.21]). In addition, for every increase in AUDIT‐C score by 1, the odds of being prescribed a medicine increased 1.48 times ([95% CI 1.35, 1.65]).

## 4 DISCUSSION

To our knowledge, this is the first study to investigate prescription of alcohol relapse prevention medicines to Australian First Nations peoples. Among these 22 ACCHSs we identified consistently low prescribing rates. In total, 0.2% of patients were prescribed relapse prevention medicines in the 12‐month reference period. This included only 3.4% of those whose AUDIT‐C scores suggested likely dependence. This finding is consistent with estimates that 2.7‐3% of those with alcohol dependence in the general Australian population received relapse prevention scripts in 2011–2012 [22]. In our study, patients were more likely to be prescribed a medicine if they were male, middle‐aged (35–54 years), attending an urban service or were screened with AUDIT‐C. Predictors of prescription identified in the present study, may assist in future research and inform clinical practice for frontline staff. Given the small percentage of likely dependent patients who received a medicine, future research should investigate barriers to prescription in this setting.

The low prescription rate of relapse prevention medicines in our sample could be due to a range of factors, including: under‐detection of dependence, GPs not offering patients a prescription to eligible patients or patients declining this treatment.

### 4.1 Likely under‐detection of dependence

The prevalence of alcohol dependence detected in our patient sample was 1.6% (AUDIT‐C score ≥9). This is slightly lower than in a recent First Nations Australian community sample (2.2%; an urban and remote site). That study used an interactive iPad tool, validated among First Nations Australians, to ask three questions on dependence (derived from International Classification of Diseases, 11th Revision, features) [14]. Our prevalence of 1.6% is close to the 12‐month prevalence of 1.4% within the general Australian community (Diagnostic and Statistical Manual of Mental Disorders, fourth edition; diagnostic interview schedule) [15]. The true prevalence of alcohol dependence in our services could be higher, given the low AUDIT‐C screening rate (17.2%) during the 12‐month period.

Over half of all patients screened had AUDIT‐C scores in the risky category (≥3 females, ≥4 males). That prevalence is far higher than the prevalence of drinking above recommended limits in the wider First Nations Australian population (20–34% individuals at high single‐occasion and life‐time risk, respectively) [37]. This could suggest that staff were more likely to screen patients whom they suspected could be risky drinkers.

AUDIT‐C was only introduced as a national key performance indicator for Aboriginal and Torres Strait Islander primary care services by the Australian Government 3 months before the baseline period ended [38]. In the overarching trial, from which we have drawn our data, AUDIT‐C screening rates have since increased in both the early intervention and wait‐control services [39].

Another reason to suspect under‐detection of dependence, is the intermittent drinking patterns that have been reported among First Nations Australians (e.g., drinking only during mourning periods [Sorry Business] or celebrations) [40]. While such patterns can occur in any setting, some participating remote services were in ‘dry communities’ where the selling and consumption of alcohol are prohibited. Heavy drinking may occur on the outskirts of dry communities [41] or on visits to nearby towns [42, 43]. Intermittent heavy drinkers can still be dependent but not readily detected by AUDIT‐C, whose questions on drinking frequency assume a regular pattern [40].

The patient, GP and service‐based factors that contribute to under‐detection of alcohol dependence in general primary care settings [19] can be compounded for First Nations populations. For example, First Nations Australians may be more reluctant to disclose risky drinking because of experiences of institutional racism [44, 45] or fear of child removal [46]. Patients from dry communities may fear legal or social repercussions if they report drinking, particularly if drinking is occurring within the community [41]. Sensitive and periodic screening by GPs may help to break down these barriers and increase earlier detection of risky drinking, including dependence.

GP time pressures are a recognised constraint on their alcohol screening [47]. These pressures can be greater in an ACCHS, as First Nations patients typically have a higher prevalence of morbidities than non‐Indigenous Australians. In some ACCHSs, other staff (e.g., nurses, Aboriginal health workers or Aboriginal health practitioners) complete AUDIT‐C screening prior to the patient seeing the GP or in health checks. If the busy GP does not notice those results or is constrained by time in their response, they may under‐detect or under‐treat dependence.

### 4.2 Not offering medicines despite detection of dependence

As well as time pressures, limited GP knowledge and confidence to address alcohol dependence have been identified as barriers to prescription in the general population [47]. Also, a potential misconception is that treating alcohol dependence is outside the scope of general practice and instead requires referral to addiction medicine specialists [48]. Furthermore, GPs treating First Nations individuals face a higher prevalence of patient comorbidities. These may include a higher prevalence of renal failure (a contraindication to acamprosate) or viral liver disease (a potential contraindication to naltrexone). In remote communities, prescribing may also be hampered by reduced access to local pathology services (e.g., to check patient suitability for a medication or to monitor progress) and lack of proximity to hospitals in the event of serious complications, particularly from a disulfiram‐alcohol reaction. Also, the medicines themselves may need to be ordered. It is not known to what extent the barriers of comorbidities or service or medication availability contributed to under‐prescribing in this sample.

Of note was the finding that just over 60% of patients in this sample received a medicine without a recorded AUDIT‐C screen. This could be due to a presentation with a very obvious and severe alcohol problem, or it could be that the prescription was a continuation of treatment that had started elsewhere (e.g. on leaving a detoxification unit).

### 4.3 Medicines potentially offered but declined

We only had information on when the medicines were prescribed, and not on when they had been discussed but declined by the patient. How GPs explain the mechanism of action, side effects, dosing schedule and out‐of‐pocket costs of the medicines are likely to impact on the patient's perception of their acceptability and value. Patients may not be aware of the medicine's role in relapse prevention. To‐date, no study has been conducted into First Nations patient perceptions of the acceptability of these medicines.

### 4.4 Cultural considerations

For many Australian First Nations peoples, the concept of ‘holistic healthcare’ extends beyond the concept of personal wellbeing to include relationships with land, family and traditional (cultural) healing practices [49]. Having a trusting therapeutic partnership where the patient and clinician have spent time developing a mutual understanding of one another is seen as the cornerstone of this care [50]. ACCHSs are leaders in this regard with many locals employed, and strong kinship and family connections to their patients [51]. Having a range of treatments available, both mainstream (Western) and First Nations cultural (e.g. flexible consultation settings and time, men's and women's groups and cultural days or camps) is likely to increase the cultural acceptability of alcohol relapse prevention medicines [50].

### 4.5 Implications for practice and research

Understanding the factors associated with prescription identified in this study may help GPs optimise care and increase prescription of the medicines where appropriate. Partnership between primary care doctors within Aboriginal Community Controlled Health Services and an addiction medicine specialist has been described as increasing GP confidence to treat alcohol and other drug use disorders [52]. Such partnerships can result in sharing of skills and knowledge, and support or referral for complex cases [52]. Community‐based initiatives, including campaigns to reduce stigma of treatment seeking could also help [17]. It will also be important to improve the health literacy about the medicines themselves among communities and individuals [53].

The present study draws on baseline data from a trial of a model of support to increase screening and care for unhealthy alcohol use. Analysis of the prescription rates post‐intervention may provide insights into ways to increase prescription of the medicines. Future research is needed to clarify the various barriers to prescription of these medicines, and approaches to increase safe and culturally appropriate use of these medications where indicated.

## 5 LIMITATIONS

While services were geographically separated, the number of discrete individuals in this sample may be slightly lower than presented, as a patient could attend more than one service. The percentage of female patients in our sample was higher than in the broader Australian First Nations population (56.1% vs. 50.7%) [54]. However, the proportion of females in our sample was only a little higher than in ACCHSs nationally (53.5% in 2016–2017) [55]. It is possible that the true number of prescriptions may vary from what was recorded in the patient software. This may occur if a prescription was written by hand rather than using the software. Also, patients may have already received a prescription from a separate service (e.g., a specialist drug and alcohol service), and so not have needed one from the ACCHS. We do not know how many eligible patients had contraindications to the medicines, or were offered the medicines but declined them.

## 6 CONCLUSION

For Australian First Nations peoples, alcohol dependence occurs in the context of the ongoing impacts of colonisation. Safe, effective and accessible treatments are needed for healing and recovery. Relapse prevention medicines are one such option. However, in the 22 participating ACCHSs, the medicines were prescribed to only a very small proportion of patients who had a likely dependent AUDIT‐C result. Patient and service‐level factors were shown to significantly predict the likelihood of prescription in our sample. These findings may be useful for GPs with both First Nations and non‐Indigenous patients who wish to increase medicine uptake for eligible patients. The findings are likely to be useful to primary care doctors working with First Nations patients in similarly colonised countries. The barriers to prescription of these medicines in ACCHS and First Nations contexts require further study.

## AUTHOR CONTRIBUTIONS

**Gemma Purcell‐Khodr:** Conceptualisation, formal analysis, original draft preparation, writing, review and editing. **James H. Conigrave:** Software, data curation, methodology, analysis support and comments on the manuscript. **K. S. Kylie Lee:** Conceptualisation and comments on the manuscript. **Julia Vnuk:** Comments on the manuscript. **Katherine M. Conigrave:** conceptualisation, comments on the manuscript and scientific integrity of the study.

## CONFLICT OF INTEREST STATEMENT

The authors declare no conflict of interest.
